# Smart Materials Leading to Restorative Dentistry: An Overview

**DOI:** 10.7759/cureus.30789

**Published:** 2022-10-28

**Authors:** Labdhi M Maloo, Aditya Patel, Sumeet H Toshniwal, Ashutosh D Bagde

**Affiliations:** 1 Department of Conservative Dentistry and Endodontics, Sharad Pawar Dental College and Hospital, Datta Meghe Institute of Medical Sciences, Wardha, IND; 2 Department of Research, Jawaharlal Nehru Medical College, Datta Meghe Institute of Medical Sciences, Wardha, IND

**Keywords:** biomaterials, bioactive materials, smart materials, smart dentistry, biosmart dental materials

## Abstract

Dental material has emerged in the last few decades with enhanced biological properties. The prime characteristics required for dental materials are that they should be compatible with oral cavity fluids such as saliva and gingival crevicular fluids. Their functionality should be enhanced in the presence of such biological factors. Scientific advancements in exploring innovative materials have led to the possibility of achieving beneficial results by using materials that respond more dynamically to the environment in which they are placed. Currently available dental materials are improvised. Restorative materials such as smart composites, smart ceramics, compomers, resin-modified glass ionomer, amorphous calcium phosphate (ACP)-releasing pit and fissure sealants, and other smart burs and orthodontic shape memory alloys have all benefited from the usage of smart materials in dentistry.

## Introduction and background

According to traditional thought, it was believed that for a material to stay for a long period of time in a patient's mouth, the material needs to be passive in nature; thus, conventional materials such as amalgam, composite, and cement were used widely [1]. The potential of materials employed in the oral cavity to sustain without interacting with the oral environment at the cellular level was typically a criterion. These materials gave good results but were not actively playing a role in the oral environment [2].

Currently, the criterion on the basis of which the material is judged has been changed. Many of the materials in materials science are functional; that is, they must perform tasks and undergo purposeful modification. They are actively involved in the operation of the structure [2]. Bioactive material is one of the most longtime effective and reliable materials. There has never been a single substance in dentistry that has been ideal in nature and met all of the requirements of a superlative material; thus, in search of ideal materials, smart materials are introduced, which may lead to smart dentistry [3].

If we consider conservative dentistry, any material is deemed to be "smart" in nature if it can sustain the remaining structure of the tooth to the point where cavity preparation can be practiced in the least invasive manner possible and can thus sustain as much tooth structure as possible by actively acting in curing process (bacteriostatic or bactericidal) [4]. A key feature of sensible behavior includes the capability to return to the first state after the input has been removed. The variety of stimuli is often pH, temperature, electricity, moisture, stress, chemical or medical specialty agents, and magnetic fields [5]. The materials researched for the sake of dentistry that actively participate in this process of correction are described as smart materials. The aim of the current review article is to provide clinical information about the chairside available smart materials and to compare their biological and functional properties, which will be useful to improve the efficacy of dental practice.

## Review

There is continuous research going on to develop advanced material in dentistry. In the current article, we have overviewed some of the smart materials that would be beneficial for both patients and the dentist.

Properties of smart materials

Dental smart materials are classified in Table 1.

**Table 1:** Classification of dental smart materials GIC: glass ionomer cement; ACP: amorphous calcium phosphate; NiTi: nickel-titanium [6]

Passive smart material	Active smart material
These materials show reaction toward the external environment without any control over it	These materials utilize the feedback loop mechanism
Composites, GIC, resin-modified GIC, and compomers	Smart composites; smart GIC; smart ceramic; pit and fissure sealant, which has fluoride-releasing ability; ACP-releasing pit and fissure sealant; self-healing composite; NiTi rotary system; smart seal obturator system; smart preparation burs

Smart materials show changes in their surroundings and react in a predictable way (Table 2).

**Table 2:** Properties Of smart materials NiTi: nickel-titanium; ACP: amorphous calcium phosphate

Properties	Description
Piezoelectric	A current is generated when mechanical stress is applied [7], e.g., smart ceramics and smart burs
Shape memory	This property states that the material has the property of changing the shape according to the applied pressure and regains its original shape once the pressure is released [8], e.g., NiTi rotary instruments
Photochromic	These materials show the property of color change according to changes in the environment [9]
Thermochromic	These materials show the property of altering according to temperature changes [9], e.g., smart impression material-smart alginate material
Magnetorheological	Material changes its state from fluid to solid when kept in magnetic field [9]
Biofilm formation	The formation of biofilm on the surface of the material helps to form a barrier between the environment and the surface [4], e.g., GC Tooth Mousse, Caridex, and Papacarie
pH-sensitive	They change their shape according to the change in pH [10], e.g., smart composites and ACP-releasing pit and fissure sealants

Smart materials in restorative dentistry

Smart Glass Ionomer Cement (GIC)

The similarity between the behavior of human dentin and the smart behavior of GIC is based on the ability of the gel structure to absorb and discharge solvent quickly in response to stimuli such as temperature, pH, and pressure changes [11,12]. GlCs have a thermal expansion coefficient that is similar to dental hard tissues. When exposed to moisture or heat, GIC shows little or minimum dimensional changes. However, when heated to 50°C in a dry environment, it exhibits significant shrinkage. The movement of water in and out of the structures causes this, which is comparable to the behavior of human dentin. GIC is an excellent dental material because of this feature [5]. Due to these behaviors shown by GIC, it shows better marginal adaptation. Because hot or cold foods and beverages can result in wide temperature fluctuations in the oral cavity, the materials in the cavity may expand or contract in response to heat. In most cases, thermal expansion coefficients help to recognize dimensional changes caused by change in temperature [12]. Various modifications are continuously made in constituents of GIC, which improved the mechanical properties of GIC, but bacterial adhesion on the surface of GIC is still a concern, which leads to secondary caries.

Fluoride Recharging of GIC

**Figure 1:**
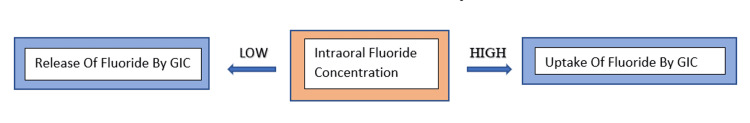
Fluoride recharging in glass ionomer cement GIC: glass ionomer cement The figure is created by the author

Another property by which GIC can be termed as smart is its fluoride-releasing ability. This property provides another advantage in preventive dentistry. The fluoride-releasing ability of GIC provides effective remineralization of incipient caries (Figure 1). When acid attack occurs, pH moves downward, which is below critical pH (i.e., 5.5); hence, fluoride is released and therefore helps in inhibiting demineralization and enhancing remineralization [13].

Casein Phosphopeptide (CPP)-Amorphous Calcium Phosphate (ACP)-Modified GIC

The increase in the consumption of aerated drinks or carbonated drinks leads to the erosion of GIC restoration and tooth material. Many modifications were made in constituents of GIC out of which one is resin-modified GIC, which is used by most of the clinicians and has been proven to be superior to the conventional GIC[14].Recently, GIC has been modified by the addition of CPP-ACP, which has been proven to increase the flexural strength of the conventional GIC. CPP-ACP-modified GIC has also shown to promote the remineralization of both cement and tooth enamel [15].

Smart Composites

Composites are currently the most widely used restorative material due to its properties and being beneficial for both the clinician and the patient, as it is tooth-colored and also has good strength. To increase its abilities further, modifications are made in composites by adding nanoparticles, ACP, and other materials, which will modify its abilities; smart composite is an alkaline, nano-filled glass restorative substance that is light-activated. It enhances the remineralization of the tooth surface when it gets demineralized by releasing hydroxyl, calcium, and fluoride ions when intraoral pH values drop below critical pH of 5.5 [16]. Smart composites are also modified to be cured in bulk thickness of up to 4 mm. It can be used in both primary and permanent teeth and is suggested for restorations in class 1 and class 2 lesions [4].

**Figure 2:**
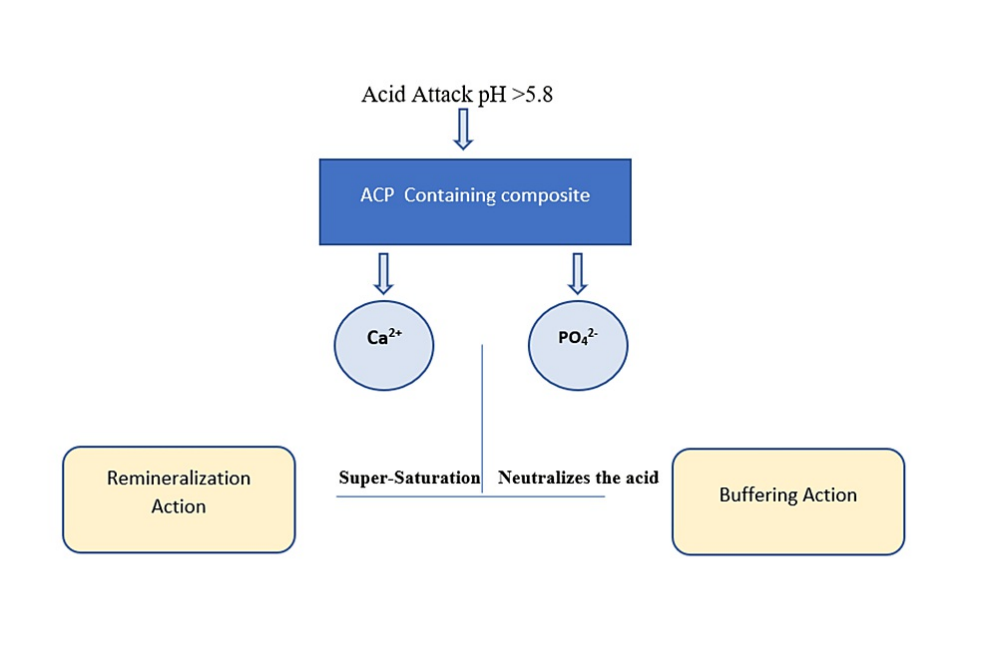
Conversion mechanism of ACP to calcium and phosphate ACP: amorphous calcium phosphate The figure is created by the author

Amorphous calcium phosphate (ACP), one of the most soluble calcium phosphates, is used in smart composites. Hydroxyapatite (HAP) is a mineral that forms the foundation of tooth enamel. Caries in a tooth is the result of exposing the oral cavity to low-pH circumstances, which can occur because of bacteria and other microorganisms that release acid, food (carbohydrate breakdown products), or acidic beverages. Caries causes a drop in pH to below 5.8, resulting in the formation of hydroxyapatite from amorphous calcium phosphate and precipitation, followed by the recovery of hydroxyapatite lost due to the acid. When the pH falls below 5.8, which is critical pH, these ions combine intraorally forming a gel in seconds, and around less than two minutes, calcium and phosphate ions are produced, and the gel converts into amorphous crystals (Figure 2) [17,18]. This property of dental composites behaves as a smart material by acting actively in the caries reduction process and also protecting the tooth structure.

Smart Ceramics

Previously, only porcelain-fused metal restoration was used as a prosthesis. There was always been a search for tooth-colored aesthetic restorations; thus, ceramic is widely used in dentistry as these restorations are metal-free and biocompatible, allowing them to merge with the natural dentition properly, and consists of feldspar, kaolin, quartz, and various oxides [19]. Developing residual compressive stress, minimizing the number of firing cycles and chemical and thermal tempering, and transforming toughening are different method to develop ceramic restoration more stronger. Ceramic restoration can be of two types, either metal-ceramic or all-ceramic. Capillary casting technique such as Captek and noble base metal such as titanium are all used in metal-ceramic restoration. All-ceramic restoration includes feldspathic or aluminum core porcelain jacket crown, complete jacket crown with leucite-reinforced core, castable ceramic such as Dicor that is 55% tetrasilic flourmica crystals, and pressable glass-ceramics. Zirconia, the newest addition to the dental ceramics family, is a polymorphic material that appears in three temperature-dependent pure forms. At ambient temperature and at about 95°C, the crystal structure is monoclinic. Zirconia transforms to a tetragonal crystal structure at temperatures above 95°C. Zirconia is a polycrystalline ceramic, which is glass-ceramics, as atoms are packed in regular crystalline zirconia-based ceramics whereas irregularly packed in glass-based ceramics. It is proved that zirconia-based ceramics are much stronger than glass-based ceramics [2]. Other advanced methods for all-ceramic restoration are slip cast technology, which is none other than glass-infiltrated ceramic, computer-aided design/computer-assisted manufacture (CAD/CAM), and copy milling. In CAD/CAM, a technician is not required; restoration is designed digitally, whereas in copy milling, restoration is designed by a technician, and digitally, it is copied to prepare. Commercially available CAD/CAM system is Cerec, Everest, and Cercon.

Cercon (smart ceramics system) was introduced 12 years ago as a CAM system for fabricating bridge frameworks and crowns in the dental laboratory. Cercon all-ceramics are now suitable for a wider range of applications, such as implant-supported Cercon frameworks and custom all-ceramic abutments, which can be utilized to restore both anterior and posterior metal and all-ceramic abutments, as well as natural-tooth preparations [19-21]. Nowadays, mica-based glass-ceramics are used in restorative dentistry. It contains a group of nonporous glass-ceramics, which is machined on mica crystals that include fluorine [21]. Great machinability and minimal abrasiveness are the two well-known advantages of it when compared to enamel [22]. The interface of mica-glassy matrix is followed by crack propagation, which is difficult because of its irregular surface. Therefore, numerous branching and crack propagation can happen, which thus guarantees machinability by preventing crack propagation [23].

Smart Nickel-Titanium (NiTi) Files

**Figure 3:**
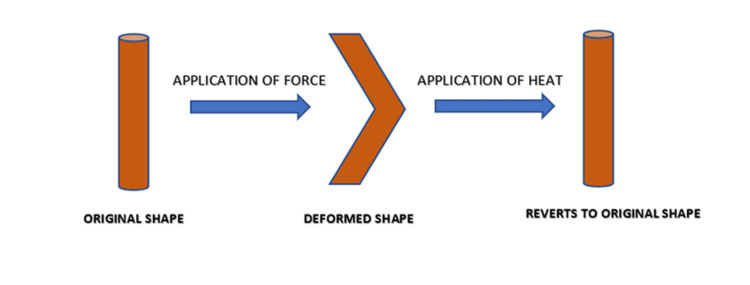
Nickel-titanium alloy showing property of shape memory The figure is created by the author

The term smart was primarily used in combination with NiTi alloys or shape memory alloys. Super elasticity and shape memory were the property because of which these were termed as smart. Along with changes in volume and density, a change in shape occurs. Superelasticity is the ability to withstand stress while returning to its original lattice shape without permanent deformation; that is, when an austenite material is deformed, it forms detwinned martensite, but it has tendency to return to its original state (spring back) and shows the property of superelasticity. The another property of shape memory refers to the NiTi file's capacity to return to its original normal shape with no deformation; that is, when detwinned martensite is heated, it forms austenite; thus, after cooling, it forms twinned martensite and therefore returns to its original shape (Figure 3) [24,25].

By applying stress or change in temperature, lattice organization can be altered. There are two phases of nitinol. First, the martensitic/daughter phase is a low-temperature phase that is body-centered cubic lattice, whereas the austenitic/parent phase is a high-temperature phase that is hexagonal lattice. During root canal treatment, NiTi files are stressed, and stress-induced transformation from austenitic state to martensitic state occurs at the speed of sound [2]. NiTi rotary instrument's superelasticity allows for easier access to irregularly shaped root canals during biomechanical preparation, with less applied lateral force and a lower incidence of the canal aberrations and transformations. When stressed at a constant temperature, nitinol changes from an austenitic crystalline-phase condition to a martensitic structure. For bending, only a little amount of force is applied, and the structure then returns to an austenitic phase, which is its original shape when the stress is relieved known as thermoelastic transformation [26]. The use of these NiTi files has made it easier and faster for dentist to achieve consistent and predictable shape through the canal than doing the same by hand instrumentation [24]. It also decreases postoperative pain for the patient.

Smart Seal Obturation System

Obturation can be described as three-dimensional filling of instrumented canals, accessory canals, and dead spaces. By obturating the material, reinfection can be prevented, hence preventing periapical infection. There are various types of canal-filling techniques used by dentists [2]. Since gutta-percha is an impermeable substance, leakage between the sealer and the dentin, as well as between the gutta-percha and the sealer, along with the presence of voids leads to failure of treatment. Various in vitro studies were conducted to check the sealing ability of gutta-percha, which showed high leak rates; thus, there was a constant search for material with better sealing properties; smart seal system is one of them. The C point system also known as smart seal system is a newly introduced point and paste system with hydrophilic polymer-based technology [27,28]. There are two main components of a smart seal: hydrophilic obturation points and a sealer. Obturation points are made up of polymorphs and are available in different tip sizes and taper. The hydrophilic property of the smart obturating material helps in absorbing the moisture and lateral expansion of the material filling voids, but consequently, a sealer should be used along with this endodontic points for proper sealing [29,30]. SmartSeal™ is available in different tip sizes and tapers (Table 3).

**Table 3:** SmartSeal™ in various tip sizes and taper ™: trademark; ISO: International Organization for Standardization The author has recreated the table from [31]

Taper	Tip sizes
6% taper	ISO tip sizes 25-45
4% taper	ISO tip sizes 25-45
ProTaper™	F1, F2, F3, F4, and F5
Sendoline™	S5 - S2, S3, and S4

Smartpaste Bio

Another example of resin-based sealant is Smartpaste Bio, which contains bioceramics [27-29]. During the setting process, Smartpaste Bio creates calcium hydroxide and hydroxyapatite as byproducts, which makes the material highly biocompatible and antibacterial. It has a delayed setting time of 4-10 hours and is hydrophilic in nature, which promotes the propoints to hydrate and swell, making them fill all the voids. The lateral forces generated are below that of the tensile strength of the dentin and low than that of the forces generated by the traditional methods [26]. Bioceramics present in the Smartpaste Bio offer dimensional stability to the sealer and make it non-resorbable in the root canal.

Smart Impression Material

Impression making is one of the foremost steps in planning treatment for the patient. Irreversible hydrocolloids and elastomeric impression materials such as polyethers and polyvinylsiloxanes are commonly used impression material in dentistry today due to their accurate impression-making properties. Still, every impression material currently used has some or the other shortcomings, and a constant search for an ideal impression material is going on. Shape memory is one of the key abilities of an impression material required to prevent distortion. Impression material must also possess thixotropic property that implies that after setting the ability, the flow of impression stops, which makes the material physically dynamic. These materials also require to have hydrophilic properties, to make void-free impression [32,33]. Some of the recently developed smart impression materials include vinylsiloxanether; it is the combination of polyether and polyvinylsiloxane; the hydrophilic property of polyether is combined with polyvinylsiloxane for proper handling in challenging situations such as moisture control and narrow gingival sulcus [34,35]. The other material is fast-set elastomeric material, which includes fast-set polyether and fast-set polyvinylsiloxane; it is used to diminish the chairside time and to enhance impression in gag reflex patients [33]. Some of the commercial examples are Imprint™ 3 VPS, Impregim™, and Aquasil Ultra (Dentsply) [36].

## Conclusions

The problems the dental fraternity is facing to achieve good clinical outcomes in patients are a major factor to initiate research in dental biomaterials so that various novel materials can be explored to gain the desired results. The introduction of smart material has revolutionized many areas in the field of dentistry, such as conservative cavity preparation; however, the constant search for an active material with multiple properties is still a requisite to achieve the desired outcomes in patients. These materials may increase the efficacy of the dental practice and also may lead to a more conservative dentistry by acting actively in the process of curing and are also beneficial for the dentist, in a way making it easy and convenient for the clinician. Thus, by implementing this most advanced class of multifunctional material, treatment quality will improve for the patient. There is no doubt that "smart materials" will hold great promise for the future.
